# Could Quantum Mechanical Properties Be Reflected on Classical Molecular Dynamics? The Case of Halogenated Organic Compounds of Biological Interest

**DOI:** 10.3389/fchem.2019.00848

**Published:** 2019-12-13

**Authors:** Lucas de Azevedo Santos, Ingrid G. Prandi, Teodorico C. Ramalho

**Affiliations:** ^1^Department of Chemistry, Federal University of Lavras, Lavras, Brazil; ^2^Department of Chemistry, Faculty of Science, University of Hradec Kralove, Hradec Kralove, Czechia

**Keywords:** halogen bonds, force-fields, molecular dynamics, non-covalent interactions, drug design

## Abstract

Essential to understanding life, the biomolecular phenomena have been an important subject in science, therefore a necessary path to be covered to make progress in human knowledge. To fully comprehend these processes, the non-covalent interactions are the key. In this review, we discuss how specific protein-ligand interactions can be efficiently described by low computational cost methods, such as Molecular Mechanics (MM). We have taken as example the case of the halogen bonds (XB). Albeit generally weaker than the hydrogen bonds (HB), the XBs play a key role to drug design, enhancing the affinity and selectivity toward the biological target. Along with the attraction between two electronegative atoms in XBs explained by the σ-hole model, important orbital interactions, as well as relief of Pauli repulsion take place. Nonetheless, such electronic effects can be only well-described by accurate quantum chemical methods that have strong limitations dealing with supramolecular systems due to their high computational cost. To go beyond the poor description of XBs by MM methods, reparametrizing the force-fields equations can be a way to keep the balance between accuracy and computational cost. Thus, we have shown the steps to be considered when parametrizing force-fields to achieve reliable results of complex non-covalent interactions at MM level for *In Silico* drug design methods.

## Introduction

Biological systems are huge, they change in time and they are very sensitive to *in vivo* conditions like temperature and environment (Ramalho et al., 2009; Freitas et al., 2014; Nair and Miners, 2014; Jurinovich et al., 2015). These facts are remembered every day by drug designers, structural biologists, biophysicists and many other professionals that need to study these systems (Nair and Miners, 2014). In order to overcome these barriers, many scientists opt to model their systems using the classical atomistic Molecular Dynamics (MD) simulation method.

The classical MD is a computational method based on Molecular Mechanics (MM) physics and its first simulation was performed by Alder and Wainright (Alder and Wainwright, 1959) in the late ‘50s. In this pioneering work, the authors discussed the difficulties to treat the many-body problem and proposed a numerical scheme to deal with multiple interactions of particles by solving Newton's motion equations. Although Alder and Wainright gave the first spark for the beginning of classical MD, the first realistic MD simulation was performed just in 1969 (Allen et al., 1969). In this work, by the implementation of Lennard-Jones potentials (essential to describe van der Waals interactions), Dr. Rahman and co-authors successfully modeled 864 atoms of liquid argon. Over the last decades, MD modeling was refined, and many different codes have been launched. Nowadays, drug design is one of the areas that most benefits from the enormous development that the atomistic MD has acquired over the years. However, the mindset that it is difficult, unnecessary or too time-consuming to parameterize new molecules may turn to final works that mislead the real interactions. Unphysical models or catastrophic geometries with very inaccurate interaction energies can be found along with an MD simulation, especially if the modeled molecule has too many chemical functions or π-conjugations (Davis and Patel, 2010; Prandi et al., 2016; Aytenfisu et al., 2017).

Despite the great effort being made by scientific programmers to enhance the quality of classical molecular simulation techniques, much more can be done by the user to improve inter- and intramolecular interactions outcomes. It should be kept in mind that intramolecular interactions are the driving force of most biomolecular phenomena (Martins et al., 2003; Adesokan et al., 2004; Ramalho et al., 2009; Poater et al., 2011; Ben-Naim, 2012; Hongo et al., 2013; Poznanski and Shugar, 2013). They are known to be quantum chemical phenomena that go beyond the classical description of matter and, in particular cases, they cannot be understood by simple electrostatic or dispersion schemes (Ramalho and da Cunha, 2011; Esrafili and Ahmadi, 2012; Wolters et al., 2014). This is one of the greatest challenges to force-field modeling since there is no classical analog to the quantum behavior of electrons.

**Figure 1:**
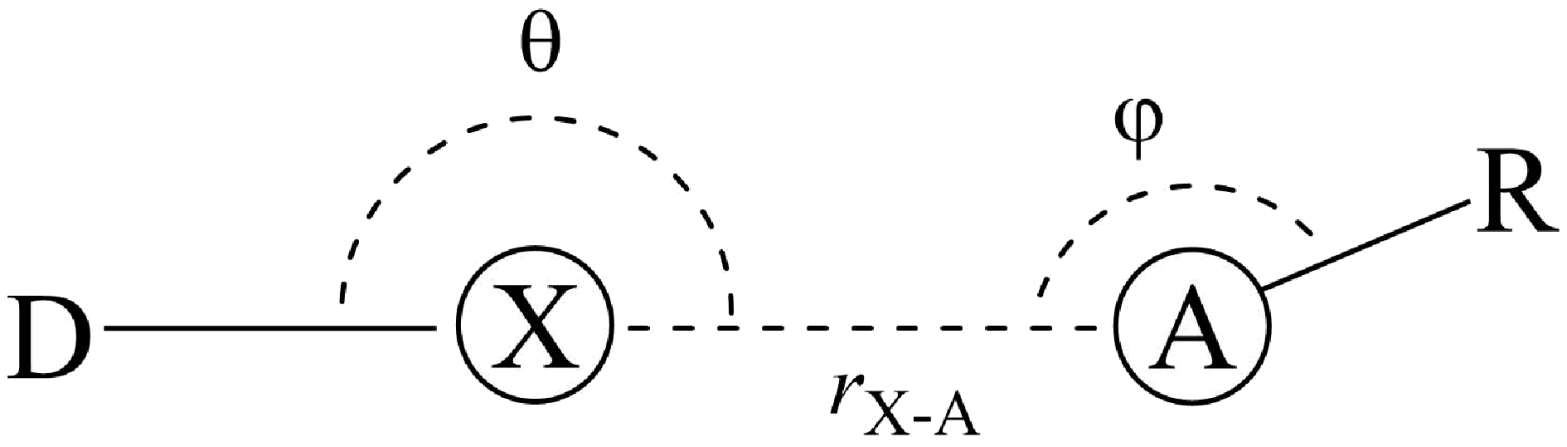
General halogen-bond scheme. The donor (D) is bonded to a halogen atom (X) that interacts with the acceptor (A) in a distance *r*.

That is the case of halogen bonds (XB), a real and relevant tool for rational drug design (Auffinger et al., 2004; Lu et al., 2009, 2010, 2012; Wilcken et al., 2013; Mendez et al., 2017). The XBs are non-covalent interactions between an acceptor (A), often Lewis base, and a halogenated molecule acting as a donor (D) (Figure 1). On one hand, some researchers address the origin of the XBs to the existence of a positive electrostatic potential region on the halogen atom (X) bond called σ-hole (Clark et al., 2007; Politzer et al., 2007, 2013; Kolár et al., 2014). On the other hand, the literature also highlights the importance of the orbital interactions, revealing the covalency part of XB (Wolters and Bickelhaupt, 2012; Wang et al., 2014; Novák et al., 2015; Wolters et al., 2015; Dominikowska et al., 2016; Bora et al., 2017; Santos et al., 2017). In contrast to molecular mechanics approaches, the XB are purely quantum chemical phenomena, whose strength grows with the size of the halogens, making chlorine, bromine, and iodine promising alternatives to promote secondary side-chain interactions inside protein cavities (Lu et al., 2010; Cavallo et al., 2016; Santos et al., 2017).

Once many compounds with biological activity have halogen atoms in their composition, the accurate description of XBs by molecular mechanics is crucial. Now, the main questions we pursue to answer are: what can we do to solve this problem?; what are the alternatives we have?; what is the best approach to build up accurate techniques to describe these interactions?

## Force-Fields: an Overview

Quantum Mechanics (QM) considers the electronic effects in molecules. On the other hand, Molecular Mechanics (MM) is based just on the interaction among classical charged particles, neglecting direct electronic effects.

Since the electronic environment around an atom changes accordingly to its neighborhood, we need an artifice to describe atoms with the same atomic numbers, but chemically different. For example, we need to distinguish the sp3 from sp2 carbons. To recover most of the electronic effects in MM based simulations different atom types should be employed. The atom types are atomic labels used to indicate chemically different atoms. In the example cited, different atom types should describe the carbons in ethanol.

After the atom types are set, the classical MD software associates each bonded or non-bonded molecular interaction to a set of parameters. In more detail, the MD software calculates the total potential (*V*_TOT_) that acts in each particle.

(1) $$\begin{array}{r} V_{TOT}\;=\;V_{S}\;+\;V_{A}\;+\;V_{D}\;+\;V_{vdw}\;+V_{C} \end{array}$$

The Equation (1) shows a generic form of a total potential: *V*_S_, *V*_A_, and *V*_D_ are the bonded terms, the stretching, angular, and dihedral potentials, respectively; and the last two terms are the non-bonded terms, in which accounts for the van der Waals interactions described by the Lennard-Jones potential (*V*_vdw_) and Coulomb potential (*V*_C_) that simulates the electrostatic interactions. It is important noticing that the terms in the total potential equation may vary depending on its implementation in the MD software. For instance, in Equation (1.1) we see the parameters $k_{\mu }^{S}$, *r*_μ_, and $r_{\mu }^{0}$, that are the stretching force constant, the length of the bond and equilibrium distance, respectively; In Equation (1.2), $k_{\mu }^{\theta }$, θ_μ_, and ${\;\theta }_{\mu }^{0}$ are the angular force constant, the angle, and equilibrium angle, respectively; The term $A_{j_{\mu }}^{SD}$ in Equation (1.3) is the dihedral torsional barrier, $n_{j}^{\mu }$ is the periodicity or the number of minima in the cosine function, δ_μ_ is the dihedral angle and $\gamma_{j}^{\mu }$ is a phase angle that represents the displacement of the dihedral angle (or torsional displacement); in Equation (1.4), ε_*ij*_, σ_*ij*_, and r_ij_ are the depth of the potential well, the distance at which *V*_vdw_ is a minimum and the distance between two particles, respectively; and finally *q* and ϵ_0_ in Equation (1.5) are the charge and the electrostatic constant, respectively. The description of each term in Equation (1) depends on these parameters and a complete set of equations together is named force-field (FF).

(1.1) $$\begin{array}{r} V_{S}=\sum_{\mu }^{N^{\underline{\text{o}}}\;bonds}k_{\mu }^{S}{\left(r_{\mu }\;-\;r_{\mu }^{0}\right)}^{2} \end{array}$$

(1.2) $$\begin{array}{r} V_{A}=\sum_{\mu }^{N^{\underline{\text{o}}}\;angles}k_{\mu }^{\theta }{\;\left(\theta_{\mu }\;-{\;\theta }_{\mu }^{0}\right)}^{2}\; \end{array}$$

(1.3) $$\begin{array}{r} V_{D}=\frac{1}{2}\;\sum_{\mu }^{N^{\underline{\text{o}}}\;Sdihedrals}\sum_{j=1}^{N^{\underline{\text{o}}}\;\cos_{\mu }}A_{j_{\mu }}^{SD}\left[1+\cos\left(n_{j}^{\mu }\delta_{\mu }-\gamma_{j}^{\mu }\right)\right] \end{array}$$

(1.4) $$\begin{array}{r} V_{vdw}=\sum_{i}^{N^{\underline{\text{o}}}\;vdw\;interactions}\sum_{i<j}^{N^{\underline{\text{o}}}\;vdw\;interactions}4\varepsilon_{ij}\left[{\left(\frac{\sigma_{ij}}{r_{ij}}\right)}^{12}-\;{\left(\frac{\sigma_{ij}}{r_{ij}}\right)}^{6}\right] \end{array}$$

(1.5) $$\begin{array}{r} V_{C}=\sum_{i}^{N^{\underline{\text{o}}}\;Coul.\;interac.}\sum_{i<j}^{N^{\underline{\text{o}}}\;Coul.\;interac.}\frac{q_{i}q_{j}}{4\pi \epsilon_{0}r_{ij}} \end{array}$$

In the last decades, the use of MD has been expanded to different areas, being necessary the creation of parameters to describe a huge set of molecular interactions at the same time or, at least, those more relevant to a certain purpose. Thus, large groups of transferable parameters have been created aiming to describe chemically similar molecules. Nowadays, there are many sets of specialized parameters for the description of many different molecular groups like polymers, proteins, solvents, small organic molecules, etc. (Jorgensen et al., 1996; Schuler et al., 2001; Wang et al., 2004; Vanommeslaeghe et al., 2010; Dickson et al., 2014).

Due to the wide use of classical MD for protein modeling, here we may highlight two of the most used sets of parameters for biomodelling: AMBER (Assisted Model Building with Energy Refinement) (Case et al., 2014), created by Peter Kollman and his group at the University of California, and CHARMM (Chemistry at Harvard using Molecular Mechanics) (Vanommeslaeghe et al., 2010), initially developed by Martin Karplus and co-workers at Harvard University. Over the years, CHARMM has expanded and gained new specific parameters for the modeling of smaller molecules.

Other diffused family of parameters for biomolecular systems are OPLS and GROMOS. The OPLS (Optimized Potentials for Liquid Simulations) (Jorgensen et al., 1996) force-field was developed by Jorgensen's group to simulate proteins in solution. In 1976, GROMOS (GROningen MOlecular Simulation) (Schuler et al., 2001) started to be developed at the University of Groningen. Originally created for biomolecules modeling, until today it is constantly updated for many different classes of molecules.

Another example of a set of parameters specially designed for small and medium-sized organic molecules is the MM*n* (*n* = 1, 2, 3, 4) family of parameters developed by Allinger and coauthors (Allinger et al., 1971, 1989, 1996; Allinger, 1977; Lii and Allinger, 1989a,b; Nevins et al., 1996; Langley et al., 2001; Langley and Allinger, 2002).

With the expansion of the use of MD simulations in the pharmaceutical field, the development of a set of parameters for drug design research was urgent. Thus, in 2004, the GAFF (General AMBER Force Field) (Wang et al., 2004) family of parameters was specifically created and tested for pharmaceutical purposes. In order to guarantee a great transferability, many GAFF equilibrium parameters were extracted from the average of X-ray and *ab initio* calculations of different molecules. Besides, pure GAFF is not yet able to model the major part of metallic interactions in complexes and can poorly describe halogen bonds (Rendine et al., 2011; Li and Merz, 2017).

## Parameterization: the Key to Realistic Results

**Figure 2:**
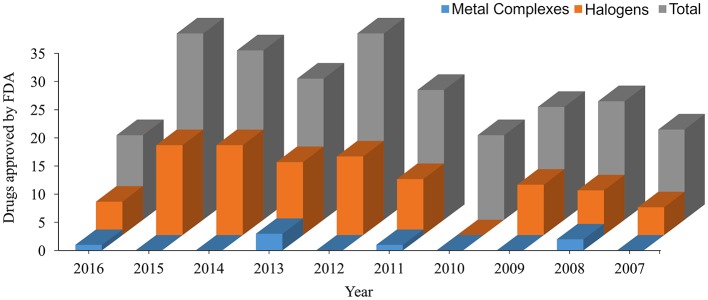
New Molecular Entity (NME) approved by the US Food and Drug Administration (FDA) in the last 10 years. All data are taken from https://www.fda.gov.

In the last decade, the US Food and Drug Administration (FDA)^1^ approved more than 230 New Molecular Entity (NME) drugs. Almost 42% of the new non-biological approved drugs contain halogen atoms, and more than 3% are metallic complexes (see data in Figure 2). These data show the importance of a specific parameterization for new drugs since most general FFs are not able to describe with high accuracy those bonds for molecular dynamics simulations (Santos et al., 2014, 2017).

Unfortunately, specificity and transferability usually have an inversely proportional relationship. Due to their good transferability, GAFF and other general force-fields are ideal to describe molecules that are indirectly involved in the studies that we would like to do. However, for very specific cases, sets of general parameters are not enough to model physical structures or interactions and we need to remodel them.

Then, theoretical scientists have realized that molecular models need to be accurate to perform a realistic simulation. For this reason, many tools were developed aiming more straightforward paths to parameterization. The greatest part of methodologies is based on the extraction of equilibrium distances, angles and dihedrals from a QM optimized structure and the force constants are derived from the diagonalization of the Hessian matrix (extracted from a QM calculation). Some examples of tools that help computational scientists to parameterize their molecules are the following: Automated Topology Builder (ATB) (Malde et al., 2011), Paratool (Mayne et al., 2013), and Joyce (Barone et al., 2013).

More specifically, ATB is much more than an on-line tool to build biomolecular force-fields for MD or Monte Carlo simulations, it can also calculate free energies and predict hydration free enthalpies. This website is very user-friendly, does not require any installation procedure and sends an e-mail to the user when the parameters are ready.

Paratool is a plugin of the software Visual Molecular Dynamics (VMD) (Humphrey et al., 1996). It was specifically developed to build parameters in CHARMM or AMBER format. It is not as automated as ATB, but it is very user-friendly since it is linked to the VMD graphical interface.

Joyce is a software specially developed to assist the derivation of parameters in GROMACS (GROningen MAchine for Chemical Simulations) (van der Spoel et al., 2005) or Moscito (Paschek and Geiger, 2002) format for MD atomistic simulations. It is also a very versatile and flexible program, in which the user can symmetrize molecular groups, set dependencies between parameters and even impose specific values to the parameters.

The three aforementioned tools are just some examples of how a specific set of parameters can be derived. They were cited in ascendant order of time-consuming and effort to create a new specific FF. The choice of modeling a molecule with an FF created in a very automated way or a much more fitted one depends on the molecule, the required accuracy and how dependent the studied property is from the molecular geometry. However, another issue that cannot be neglected is the more complex intermolecular interactions, such as the halogen bonds. The difficulties of modeling intramolecular parameters are beyond the simple extraction and fitting of the parameters: they are also led by the MD software limitations.

Some MD software like AMBER do not distinguish intra from intermolecular parameters for van der Waals and Coulombic charges. Although MD simulations may give good results for many physical and macro properties of a large number of different systems, many times specific micro-interactions are not modeled in a refined way. This is the case of some vibrational modes: even if a very precise parameterization is done, coupled vibrational modes in very conjugated molecules are extremely hard to describe (Prandi et al., 2016; Andreussi et al., 2017). The difficulty of an accurate description is mirrored in the fact that most MD simulation programs do not couple molecular motions like a stretching mode with an angular bending or the stretching modes of two adjacent atom pairs (Andreussi et al., 2017; Cerezo et al., 2018). More precisely, all mentioned terms in Equation (1) are expressed as sums of contributions, each one depending on a single internal coordinate. In this way, the off-diagonal terms (or hybrid terms) of the Hessian are not explicitly taken into account. Here, it is important to emphasize that it is mathematically and physically possible to derive parameters considering the cited couplings (Cerezo et al., 2018), but the implementation of a force-field functional form that describes the coupled terms can still not be done in many MD software.

It is evident that neither the best set of parameters can completely recover the electronic effects of a given molecule along an MD trajectory. Although some MD simulation programs are starting to be more flexible in terms of a force-field functional implementation, like GROMACS and Moscito (Cerezo et al., 2018). There is a still long path to achieve the full force-field functional form flexibility. Indeed, the maximum refinement that a normal user of mostly MD programs can do is to construct his own set of parameters. However, diving into very specific cases simple parameterizations can still be not enough.

## Beyond the Limits

As defined in the introduction, the halogen bonds (XB) are non-covalent interactions between the halogen bond donor (D) and the halogen-bond acceptor (A) (Figure 1). Thus, the force-fields will describe these phenomena through the van der Waals term (Equation 1.4) and by the Coulombic term (Equation 1.5).

**Figure 3:**
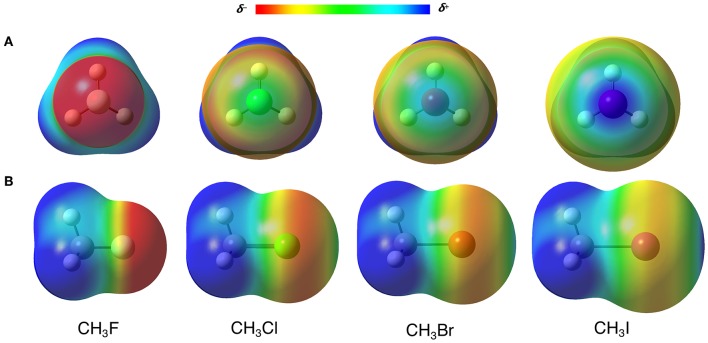
**(A)** Front and **(B)** side view of the electrostatic potential surfaces (at 0.02 a.u.) from −0.3 (red) to 0.3 (blue) a.u. of CH_3_X (X = F, Cl, Br, I) molecules. Computed at B3LYP-D3(BJ)/def2-TZVP, using Gaussian 09 (Frisch et al., 2009).

Many researchers address the origin of the XBs to the σ-hole, classifying them as σ-hole interactions (Clark et al., 2007; Politzer et al., 2008). The σ-hole is a positive region on the electrostatic potential surface (ESP), that arises from a charge anisotropy effect along with the D–X bond (Clark et al., 2007; Politzer et al., 2013). In other words, the electron density polarizes toward the D–X, generating an electron depletion in the back of the halogen (X) toward the D–X bond axis (see the blue regions over the halogens in Figure 3, in which D = CH_3_) (Politzer et al., 2012). For the σ-hole model, the strength of the XBs, which increases along X = F < Cl < Br < I, is directly correlated to the increase of the positive electrostatic potential on the halogen. In this sense, to perform a classical FF description of the XBs, the attraction between A and X should be rigorously described by the Coulomb potential. However, here we have at least two barriers to overcome: firstly, the XB cannot be seen as the attraction of two points charges as described by Equation (1.5), but the interaction of two densities; secondly, even using the point charge scheme, halogen atoms often have negative charges that would cause an electrostatic repulsion between X and A, not allowing the XB to happen. The fact is, something totally different from the usual parameterization must be done.

**Figure 4:**
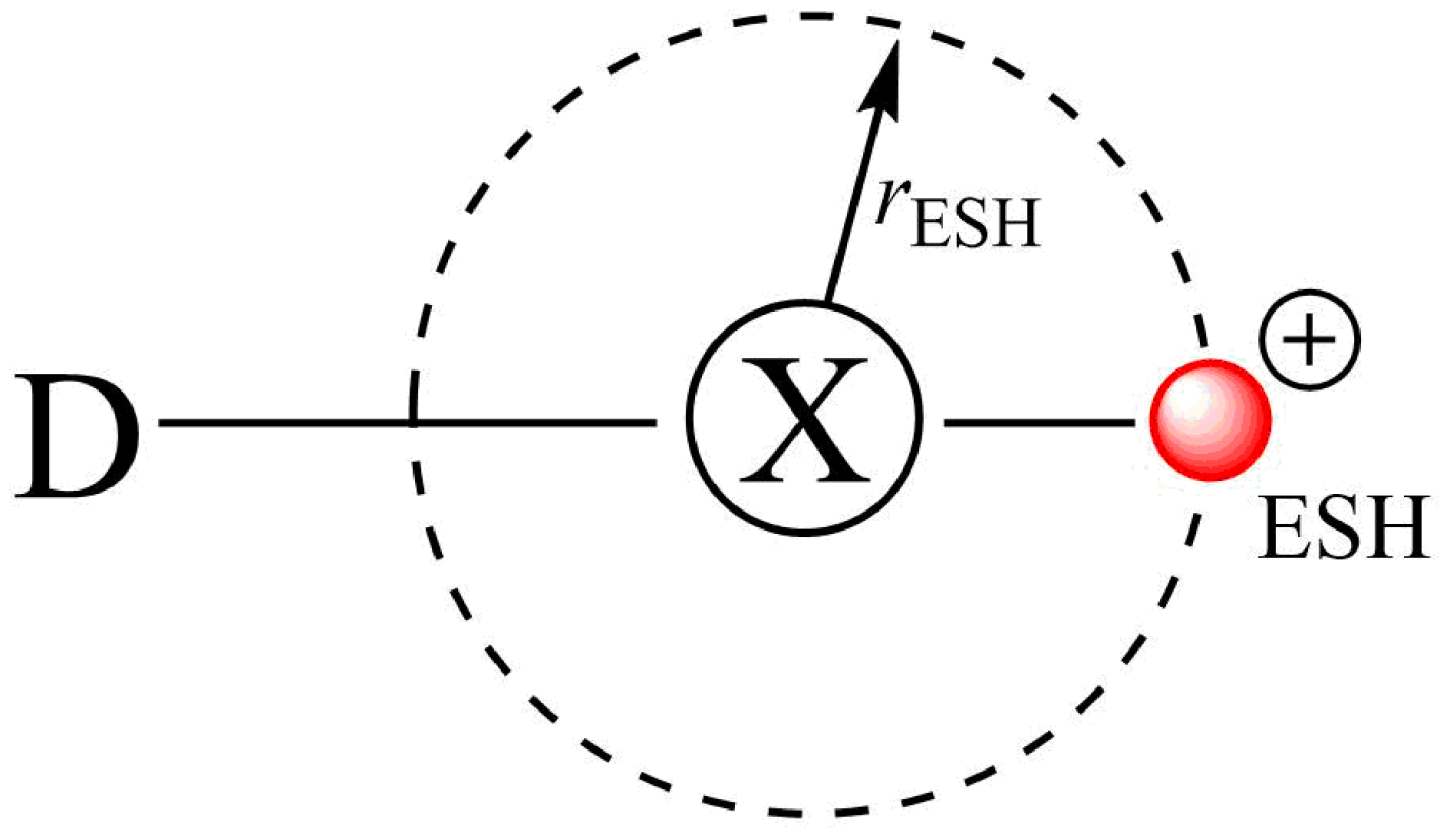
The explicit σ-hole (ESH) scheme to model halogen bonds via the molecular mechanics approach.

The first attempt to describe the XB through molecular mechanics was suggested by Ibrahim who introduced the Explicit σ-hole (ESH) theory (Ibrahim, 2011, 2012; Kolár et al., 2013). The ESH is a way to model the σ-hole as a massless positive point charge bonded to the halogen atom at a certain distance (*r*_ESH_) (Figure 4). In general, there are two parameters to be fitted: the charge of the massless point and its distance to X (*r*_ESH_).

The ESH strategy has promoted huge advances for the modeling of XB in a biological environment predicting energy minima points in halogen-bonded systems along the potential energy surfaces. However, it is totally based on the classical electrostatic point of view of chemical interactions, that is, the electrostatic attraction between two point charges.

**Figure 5:**
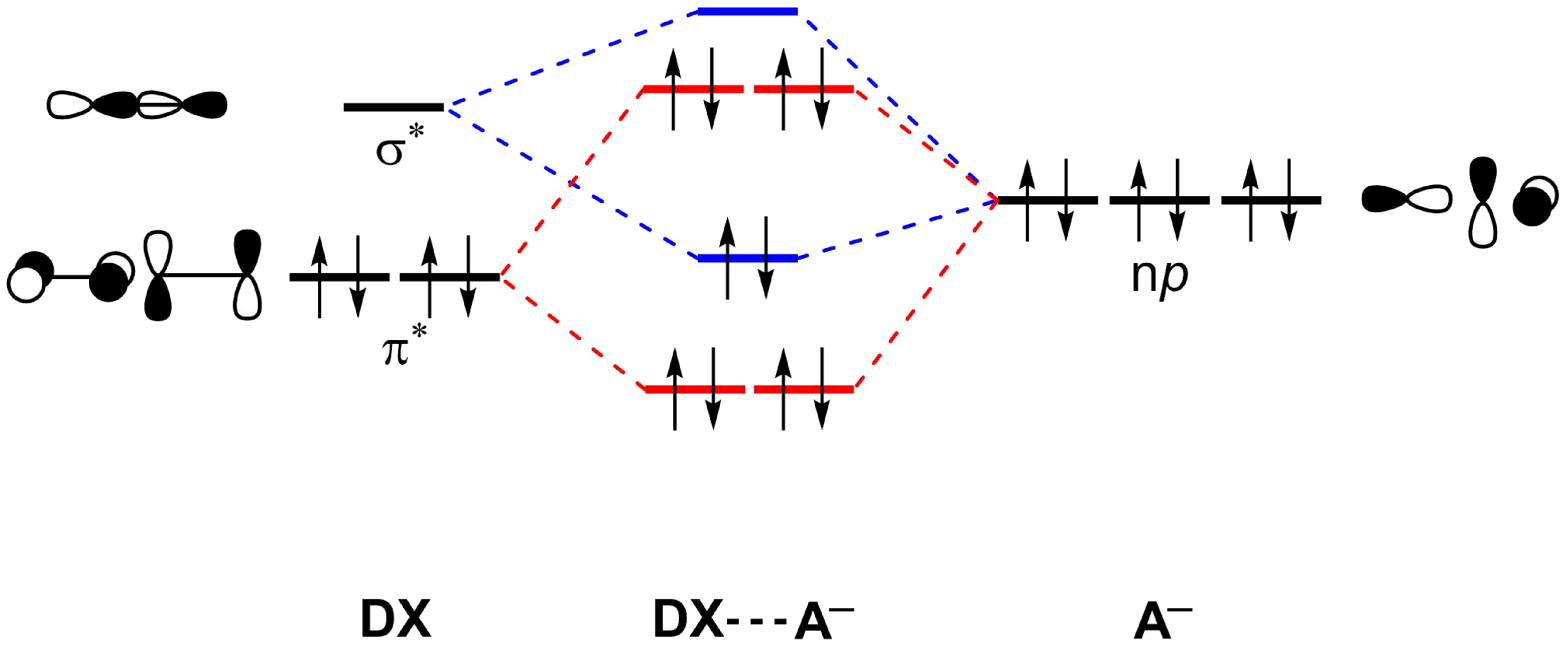
Simplified molecular orbital perspective of halogen-halide bonds. Main attractive interactions (blue) and repulsive interactions (red) are highlighted. X, D, A = F, Cl, Br, I.

In fact, the XBs are a mix of attractive dispersion, electrostatics and orbital interactions in balance with repulsive orbital interactions (Pauli repulsion) and should not be described neglecting either one of them (Huber et al., 2013; Wolters et al., 2014; Santos et al., 2017). Figure 5 shows a simplified scheme of the halogen-bonding mechanism by Wolters and Bickelhaupt in the sight of Kohn-Sham density functional molecular orbital theory (Wolters and Bickelhaupt, 2012). An occupied molecular orbital of the acceptor, described by n*p* orbitals of the halides, interacts with an unoccupied molecular orbital of the halogenated molecule (DX) to promote an attractive orbital interaction. Here, the doubly occupied orbital can be further extrapolated to any doubly occupied MO. See that the unoccupied molecular orbital of the DX molecule will have a strong sigma anti-bonding orbital ($\sigma_{\text{D}-\text{X}}^{*}$) character. The Pauli repulsion originates from the interaction between the doubly occupied orbitals.

In practice, the σ-hole model often seems to work, but just by coincidence. In previous work, we have shown that the maximum ESP values on σ-hole (*V*_max_) and the unoccupied orbital which contains the contribution of $\sigma_{\text{D}-\text{X}}^{*}$ may have a similar origin (Santos et al., 2017). In other words, by increasing the value of *V*_max_, the $\sigma_{\text{D}-\text{X}}^{*}$ will be stabilized and the interaction energy will become more stable.

Once the XBs have an important contribution of non-classical interactions, they cannot be described only by the Coulomb potential to get the ideal parameterization. In the traditional FF equation (Equation 1), the other alternative is to look at the van der Waals term. The Equation (2) is the Lennard-Jones 12-6 potential (Lennard-Jones, 1931) written in a different way than in Equation (1.4). Here, the positive part is the repulsion term and the negative part is the attractive term. The parameters are reduced to ε, the potential energy depth, and *r*_*e*_, the equilibrium distance.

(2) $$\begin{array}{r} V_{vdw}=V_{LJ\;12-6}=\varepsilon \left\{{\left(\frac{r_{e}}{r}\right)}^{12}-{2\left(\frac{r_{e}}{r}\right)}^{6}\right\} \end{array}$$

In theory, the repulsive part of Equation (2) would account for the Pauli repulsion, which is decently described by the traditional FF being the result of the steric hindrance between two atoms. The attractive term would account for the dispersive and orbital interactions. The first is also quite well-parametrized but the same cannot be said for the orbital interactions (Wu et al., 2012; Santos et al., 2014). The main problem of neglecting the orbital interactions in molecular mechanics is to get overestimated destabilizing energies at low range distances (Santos et al., 2014, 2017). At this interaction bond length, the Pauli repulsion and charge transfer are exponentially intensified.

One way to minimize this problem is to use the LJ 10-6 potential (Equation 3). With a lower exponential factor in the repulsion term, the interaction energies at low range distances are less destabilized (Du et al., 2013; Santos et al., 2014, 2017). However, the LJ 10-6 does not bring geometric improvements in comparison with the LJ 12-6, mainly in the cases that the non-covalent interactions are extremely directional, as the halogen bonds. The Lennard-Jones potential can model if a Lewis base will approximate toward the σ_D−X_ bond axis or not and how it would affect the interaction energy (Soteras Gutiérrez et al., 2016; Bernardes and Canongia Lopes, 2017).

(3) $$\begin{array}{r} V_{vdw}=V_{LJ\;10-6}=\varepsilon \left\{{\left(\frac{r_{e}}{r}\right)}^{10}-{2\left(\frac{r_{e}}{r}\right)}^{6}\right\} \end{array}$$

## Changing the Potential Equations

If the actual model does not work for a specific system, we must reformulate it. So, why not do the same for FF equations? Nevertheless, it is not necessary to build an equation from scratch. Wiser is to modify a well-known model. In the case of reformulating new non-bonded force-field terms, subtly modifications into the *V*_*C*_ or *V*_*LJ*_ have been done to get more accurate functions.

The directional dependence of halogen bonds can be understood by looking at the σ-hole and MO theories together. The interaction angle θ must be close to 180° to lead the interaction toward the D–X bond axis (Figure 1). This is the geometry configuration that would maximize the electrostatic and orbital interactions for both σ-hole and MO theories (Riley et al., 2009, 2013; Esrafili and Ahmadi, 2012; Santos et al., 2017). The angle *φ* depends on the electronic structure of the acceptor (A) in order to maximize the attractive donor-acceptor orbital overlaps (see Figure 5). For instance, having an *sp*^2^ oxygen as acceptor, *φ* would be around 120° to provide the frontal overlap between the lone pair of the oxygen (LP_O_) and the $\sigma_{\text{D}-\text{X}}^{*}$ orbital. By the same perspective, for an *sp* nitrogen as acceptor, *φ* would be around 180° and, for π acceptors, *φ* would be around 90° (Cavallo et al., 2016; Nziko and Scheiner, 2016; Santos et al., 2017).

In a very clever way, Carter and co-workers (Carter et al., 2012; Scholfield et al., 2015; Koebel et al., 2016) have introduced the angular dependence into the LJ 12-6 and Coulombic potentials to describe bromine bonds, which was later extended to chlorine and iodine. The Equations (4) and (5) are the *ff* BXB functions to describe the non-bonding terms of XBs. The effective halogen charge (*Z*_x_) is defined by the amplitude (*A*) and the baseline (*B*) of the cosine function, which has the period ν and α = 180 − θ. In *V*_LJ_, 〈*r*_*vdw*(*X*)_〉 is now the average radius of the bromine at the energy minimum.

(4) $$\begin{array}{r} V_{C}=\frac{Z_{X}Z_{A}e^{2}}{Dr^{n}}\;;\;Z_{X}=A\cos\left(\nu \alpha \right)+B \end{array}$$

(5) $$\begin{array}{l} V_{LJ}=\sqrt{\varepsilon_{X}\varepsilon_{A}}\{{\left(\frac{r_{vdw(A)}+\langle r_{vdw(X)}\rangle -\Delta r_{X}\cos\left(\nu \alpha \right)}{r}\right)}^{12}\\ \;-2{\left(\frac{r_{vdw(A)}+\langle r_{vdw(X)}\rangle }{r}\right)}^{6}\} \end{array}$$

Parameterized to predict the halogen bonds in DNA junctions, the variation in the interaction energies were ~0.06 to ~0.7 kcal.mol^−1^ in comparison to the experimental data. The *ff* BXB functions also give good values of θ, from ~146 to ~122°.

Du et al. have introduced new polarizable non-bonded functions to the force-field equations in order to reproduce the XBs, the PEff model (Du et al., 2013). The electrostatic potential is defined by (6), in which *Q* is a constant, α, β, and ζ are parameters from *ab initio* electrostatic potential, *r*_1_ is a coordinate in the equatorial area, *R* is the distance from the halogen atom toward the D–X axis and *r* is the halogen-bond length.

(6) $$\begin{array}{r} V_{elst}\left(r_{1},R,r\right)=Q\cdot \left[\exp\left(-\alpha r_{1}-\beta R\right)-exp\left(\zeta r\right)\right]\;/r \end{array}$$

The Lennard-Jones potential was used to simulate the repulsion and dispersion interactions (7), in which *r*_e_ is a function of θ, *r*_e, T_ is the transverse distance, *r*_e,L_ is the longitudinal distance and λ is a steepness parameter manually set to 1.26.

(7) $$\begin{array}{r} V_{rd}=4\varepsilon \left\{{\left(\frac{r_{e}\left(\theta \right)}{r}\right)}^{10}-{\left(\frac{r_{e}\left(\theta \right)}{r}\right)}^{6}\right\}\;;\;\\ \begin{array}{c} r_{e}\left(\theta \right)=r_{e,T}sin^{2}(\lambda \theta )+r_{e,L}cos^{2}(\lambda \theta ) \end{array} \end{array}$$

The third and last term is the polarization energy (8), in which *E* is the electronic field, *E*_tot_ incorporates the induce dipole effects and α is the isotropic polarizability of the halogen.

(8) $$\begin{array}{r} V_{pol}=-\frac{1}{2}\alpha E\cdot E_{tot}\exp\left(1.0-{\left(\frac{r}{r_{\min}}\right)}^{2}\right) \end{array}$$

The PEff functions have demonstrated a good performance in comparison with MP2 methods to predict the binding energies for chlorine, bromine, and iodine. Applied to well-known crystal structures, the deviation of the halogen-bond lengths was <0.1 Å and giving bond angles close to 180°.

Both *ff* BXB and PEff are complete force-field and already functional, albeit some tests with molecular dynamics must be carried out. Moreover, they only consider Cl, Br, and I as donors and Lewis basis with available electron lone pairs (i.e., A = S, O, N) as acceptors. Nonetheless, molecules with π orbitals can also act as halogen bond acceptors and must be considered since there are several aromatic structures in the biological environment. In this sight, a new LJ 10-6 function has been proposed that takes into account the halogen-bond acceptor nature and also includes the fluorine in the XB donor, the *E*_mod_ (Equation 10) (Santos et al., 2017). Indeed, at certain conditions regarding the electronic structure of the whole donor molecule, the fluorine atom can form strong halogen-bonded complexes, sometimes as strong as the chlorine bonds (Wolters and Bickelhaupt, 2012; Santos et al., 2017).

(9) $$\begin{array}{r} V_{LJ}=E_{mod}=\varepsilon \;\left\{{\left(\frac{r_{e+\frac{\delta cos\Theta }{6}}}{r}\right)}^{10}-{2\left(\frac{r_{e}}{r+\gamma }\right)}^{6}\right\} \end{array}$$

The *E*_mod_ empirical potential (10) has two new parameters: δ and γ (11). The parameter δ accounts for the attractive orbital interactions regarding the angular dependence to minimize the repulsion term, based on the synergy between *V*_max_ and the $\sigma_{\text{D}-\text{X}}^{*}$ energies, as aforementioned. The *V*_max_ is calculated by QM methods, α is the van der Waals radius of the halogen atom and β is a constant, in which β = 2.5 for lone pair acceptors and β = 0.432 for aromatic acceptors. The parameter γ is a function of δ to rebalance the potential.

(10) $$\begin{array}{r} \delta =\frac{\beta V_{\max}}{4\pi \alpha^{3}}\;;\;\gamma =\left[\frac{2^{2-\delta }\left(1-\delta \right)}{25r}\right] \end{array}$$

The *E*_mod_ was designed to use the *r*_e_ already parameterized by any force-field without halogen-bond corrections. In practice, *V*_max_ should be obtained by a QM calculation and used to fully define the parameters in Equation (11). Also, the *E*_mod_ could be used the general *V*_LJ_ function of the FF, since when *V*_max_ is not given (i.e., equal to zero), the parameters δ and γ will tend to zero, and this function will behave like a traditional LJ 10-6 potential. However, *E*_mod_ has not been tested with a complete force-field equation and there are no parameters for iodine.

Performing subtle modifications in the traditional empirical potentials is a good strategy to improve force-field equations. There are many other examples of modified potentials fitted to obtain reliable results of complex properties at the molecular mechanics level (Bernardes and Canongia Lopes, 2017; Franchini et al., 2018; Lin and MacKerell, 2018; Nunes et al., 2018). This approach eases the implementation of these functions by not requiring a huge effort to build a code from the beginning but using an already existing open-access and well-working code.

The use parameters obtained from previous quantum mechanical calculations can surely improve the results of a molecular dynamics simulation, but the next step is to rework the potentials in Equation (1) to further decrease the level of empiricism. That is where we find the *ab initio* force-fields (McDaniel et al., 2016; Xu et al., 2018; Pérez-Conesa et al., 2019). In principle, it would be possible to properly describe any non-covalent interactions with *ab initio* derived potentials, considering their specific properties, with a manageable computational cost.

## Summary and Outlook

Through the last decades, computational methods have been employed in the investigations around chemical properties of the matter. The evolution of technology has allowed us to go deeper into the atomic level to retrieve information about chemical bonds and non-covalent interactions. However, the computational cost has been the border of how further our knowledge could go. To overcome these borders, cheap computational approaches based on classical mechanics have emerged.

In this review, we have discussed how cheap approaches, like molecular dynamics (MD) and molecular mechanics (MM) calculations, can be improved. Toward this goal, the parameterization of their force field (FF) equations is the key. Most of the parameters can be obtained by quantum mechanical (QM) calculations together with specific tools to modify and generate more accurate FF. Nevertheless, we have further explored one case that only setting up better parameters is not enough to retrieve the real information from a non-covalent interaction.

Being purely quantum chemical phenomena, the halogen bonds (XBs) have required the replacement of some FF potentials, since simple classical equations could not describe the properties of systems they are involved in. This replacement has been wisely done by modifying and introducing new parameters to well-known potentials. The new potentials to describe XBs were fit to high-level QM calculations, showing good agreement with crystal structure data. Thus, we strongly believe that the classical mechanical approaches will evolve by introducing new potentials based on *ab initio* calculations. The scope of this review is to highlight the relevance of ab initio parameterizations if the recovering of quantum chemical effects, lost by MM simulations, is wanted.

## Author Contributions

LS and IP held the literature research and prepared the initial draft of this review. LS, IP, and TR were responsible for proofing and preparing the final copy of this review.

### Conflict of Interest

The authors declare that the research was conducted in the absence of any commercial or financial relationships that could be construed as a potential conflict of interest.

## Footnotes

^1^US Food and Drug Administration (FDA). Available online at: https://www.fda.gov/ (accessed November 4, 2017).
